# A new model for evaluating pressure-induced vascular tone in small cerebral arteries

**DOI:** 10.1007/s10237-023-01774-7

**Published:** 2023-11-04

**Authors:** Alberto Coccarelli, Sanjay Pant, Ioannis Polydoros, Osama F. Harraz

**Affiliations:** 1 Zienkiewicz Institute for Modelling, Data and AI, Faculty of Science and Engineering, Swansea University, Swansea, UK; 2 Department of Pharmacology, Larner College of Medicine, and Vermont Center for Cardiovascular and Brain Health, University of Vermont, Burlington, USA

**Keywords:** Cerebral autoregulation, Myogenic signalling, Calcium sensitization, Vascular biomechanics, Multiscale modelling

## Abstract

The capacity of small cerebral arteries (SCAs) to adapt to pressure fluctuations has a fundamental physiological role and appears to be relevant in different pathological conditions. Here, we present a new computational model for quantifying the link, and its contributors, between luminal pressure and vascular tone generation in SCAs. This is assembled by combining a chemical sub-model, representing pressure-induced smooth muscle cell (SMC) signalling, with a mechanical sub-model for the tone generation and its transduction at tissue level. The devised model can accurately reproduce the impact of luminal pressure on different cytoplasmic components involved in myogenic signalling, both in the control case and when combined with some specific pharmacological interventions. Furthermore, the model is also able to capture and predict experimentally recorded pressure-outer diameter relationships obtained for vessels under control conditions, both in a Ca^2+^-free bath and under drug inhibition. The modularity of the proposed framework allows the integration of new components for the study of a broad range of processes involved in the vascular function.

## Introduction

Several cerebrovascular pathological conditions involve the malfunctioning of the contractile apparatus in resistance blood vessels (Cipolla et al. 2014b; Good et al. 2018; Fan et al. 2022). In the brain, as well as in many other vital organs such as heart, stomach and kidneys, small arteries and arterioles can actively regulate their diameter to maintain almost constant blood flow in spite of sudden pressure changes. Besides guaranteeing adequate organ perfusion across different physiological conditions, this intrinsic regulation also protects the vascular wall against excessive mechanical stress. Pivotal in this autoregulatory mechanism is the pressure-induced generation of ‘myogenic’ tone in the vascular SMCs which drives the contraction/dilation of vessel wall (Davis 2012). Early studies (Harder 1984; Knot and Nelson 1998; Osol et al. 2002; Mufti et al. 2010) in small cerebral vessels showed that an augmentation in luminal pressure causes SMC membrane depolarization with consequent intracellular Ca^2+^ elevation. Applied mechanical forces can be sensed by the vascular wall through a group of ion channels, whose composition depends on the physiological function of the vessel (Mederos and Schnitzler 2008; Pires et al. 2017; Cui et al. 2022; Harraz et al. 2022). The pressure-induced increase of Ca^2+^ in the cytosol enhances the production of myosin light chain kinase (MLCK) that, by phosphorylating the myosin light chains (LC_20_), favours the actin–myosin interaction and consequently the generation of vascular active tone, leading to eventual constriction. However, experimental evidence (Chrissobolis and Sobey 2001; Cipolla et al. 2002) indicates that Ca^2+^ signalling is not the only cellular pathway through which pressure modulates the cell’s contractile machinery. It was shown that above certain pressure levels ($\approx$ 40–60 mmHg) the cell tone is also regulated by pathways that do not directly affect the level of intracellular Ca^2+^ but act rather as parallel mechanisms which can ‘change the sensitivity’ or ‘sensitize’ the contractile machinery to Ca^2+^ variations and re-organize the cytoskeleton. Pressure-activation of the RhoA/Rho-associated kinase (ROCK) pathway increases the cell contractility by inhibiting the activity of myosin light chain phosphatases (MLCP) which in turn increases the myosin fraction available for the cross-bridges (XBs) formation (Gokina et al. 2005; Johnson et al. 2009; El-Yazbi et al. 2010; Cole and Welsh 2011). High pressure levels also promote actin polymerization via Protein Kinase C (PKC). This cytoskeleton remodelling strengthens the connections between plasma membrane, actin and extracellular matrix which ultimately augments the transmission of contractile force generated by cross-bridges cycling (Gunst and Zhang 2008; Walsh and William 2013; Moreno-Dominguez et al. 2014; El-Yazbi et al. 2015).

To quantify processes underlying disease onset and development, and to virtually assess the efficacy of medical interventions it is mandatory to develop accurate in silico models describing cerebrovascular function. The modelling work by Yang et al. (2003a, b) represents reference studies for describing tone generation and contractility in SCAs. Due to its importance, several authors have devised complex and accurate computational methodologies (Yang et al. 2003a; Kapela et al. 2008; Edwards and Layton 2014) describing the Ca^2+^ dynamics regulating SMC tone under different physiological conditions and/or pharmacological inhibition. On the other hand, there is no established SMC contractility model accounting for the ROCK and PKC pathways, which in cerebral arteries are dominant at middle-high pressure levels. Through this work, we lay the foundation towards the realization of a holistic computational framework integrating all known signalling pathways involved in the pressure-induced tone generation. This is achieved by introducing a multi-scale model that is able, for the first time, to quantitatively describe how luminal pressure impacts all major SMC signalling pathways and ultimately the level of tissue contractility. Moreover, the developed framework can be used as an in silico platform for predicting the effect of different types of pharmacological interventions at both intracellular and tissue levels.

## Methods

The proposed model allows to evaluate how luminal pressure modulates the vascular tissue tone and consequently the inner diameter of SCAs. This framework is constituted by chemical and mechanical sub-models which are sequentially coupled. The chemical sub-model describes the pressure-induced SMC signalling, with some of its variables representing the input for the mechanical sub-model, which translates the active tone information from cellular to tissue level, with consequent diameter adaptation. Model parameter estimation is carried out in two steps by using data under control conditions and pharmacological interventions.

### Chemical sub-model

According to previous studies (Lagaud et al. 2002; Lidington et al. 2013), we assume that the pressure-induced pathways are not sequentially activated but they can independently and parallelly contribute to tone generation across wide and overlapping pressure load ranges. Inspired by the recent logic-based model by Irons and Humphrey (2020), we describe the activity level of intracellular ions and proteins involved in the pressure-dependent pathways through a signalling network. Activation/inhibition processes (connecting the network nodes) are defined by means of logistic functions. Here, each network node represents either the concentration/content of an ion/protein or the phosphorylation level of a protein, normalized with respect to the maximum value. With respect to the model by Irons and Humphrey, we introduce some changes that enable us to better capture certain features of the considered SMC signalling. First of all, a generic logistic function $\chi (\xi )$ $\in$ [0,1] (representing a intracellular process) depends on the activity level of a node (intracellular variable) $\xi$ $\in$ [0,1] via

1 $$\begin{aligned} \chi (\xi )=B_0+(1-B_0)(1+K^n) \frac{\xi ^n}{\xi ^n+K^n}, \end{aligned}$$

where $B_0$ represents the basal activity level (which does not need to be necessarily equal to 0, but still $< 1$), whilst *n* and *K* are positive coefficients of the logistic function. These three parameters ($B_0$, *n* and *K*) need to satisfy the following constraints:

2 $$\begin{aligned} \chi (0)=B_0,\quad \chi (1)=1, \end{aligned}$$

and are identified during the model parameter fitting against experimental measurements. In general, if a node ($\xi _i$) is affected by multiple independent activation/inhibition processes (i.e. $\chi _l$ and $\chi _m$) of other nodes (i.e. $\xi _j$ and $\xi _k$), the conditional operators ‘AND’ ($\wedge$), ‘OR’ ($\vee$) and ‘AND NOT’ ($\lnot$) are used:

3 $$\begin{aligned} {\xi _j \wedge \xi _k= {\chi _l}(\xi _j){\chi _m}(\xi _k),~~~\xi _j \vee \xi _k={\chi _l}(\xi _j)+{\chi _m}(\xi _k)-{\chi _l}(\xi _j){\chi _m}(\xi _k),~~~\xi _j \lnot \xi _k= {\chi _l}(\xi _j)(1-{\chi _m}(\xi _k)).} \end{aligned}$$

**Fig. 1:**
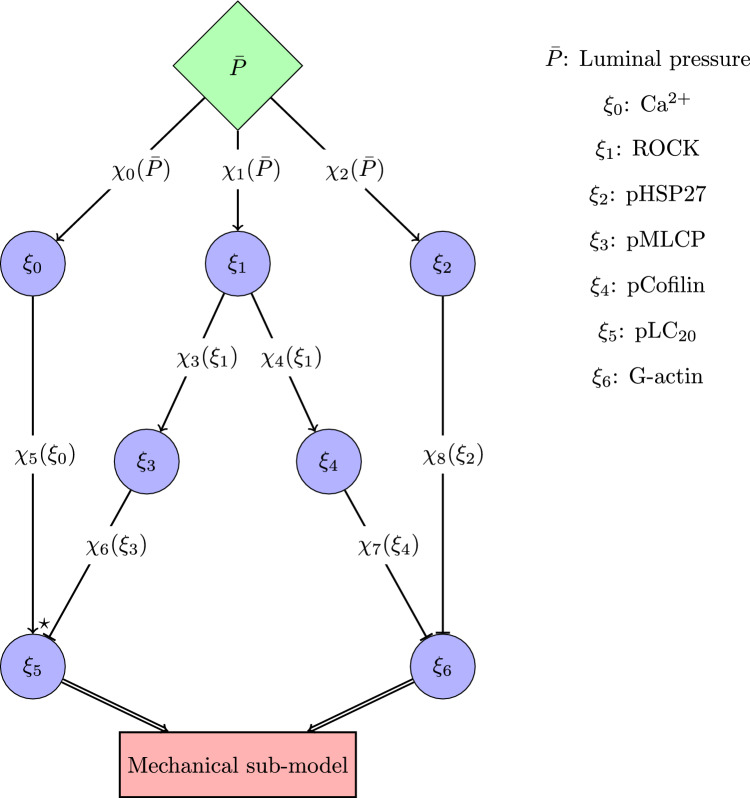
Chemical sub-model represented as signalling network. The symbols ${\leftarrow }$ and $\vdash$ represent activation and inhibition processes, respectively. The symbol $\star$ indicates that the dynamics of $\xi _5$ is described through an ad hoc equation that does not follow the logic-based rules of the remaining part of the network, whilst ${\Leftarrow }$ implies that the associated variable serves as an input for the mechanical sub-model

However, if enough information is available, the dynamics of a network node can be described through a specific ‘ad hoc’ equation that does not necessarily follow the logic-based formalism. The architecture of the intracellular network (i.e. the connections between nodes) is devised based on the current experimental knowledge/evidence on pressure-induced contractility in SCAs (Johnson et al. 2009; Cole and Welsh 2011; Moreno-Dominguez et al. 2014). Hence, we assume that the stimulus normalized pressure $\bar{P}$ (with respect to a maximum pressure $P_{\text {max}}$ beyond which the generated tone does not increase) is able to modulate the SMC signalling (and consequently the vascular tone) through three distinct pathways, involving cytosolic Ca^2+^, ROCK and PKC signalling (Fig. 1).

An increase in luminal pressure (and consequently in transmural stress) leads to a complex Ca^2+^ dynamics involving an orchestra of ionic channels which ultimately results in an intracellular Ca^2+^ concentration elevation (Jackson 2021). It is worth noticing that cytosolic Ca^2+^ increase is required for tone generation since upon extracellular Ca^2+^ removal vessels do not exhibit active behaviour (Knot and Nelson 1998; Schubert et al. 2008). Here, for the sake of simplicity, we assume that the effect of pressure on the normalized intracellular Ca^2+^ level $\xi _0$ can be resumed through an activation function ${\chi _0(\bar{P})}$, which is defined within the maximum ranges reported among different experimental studies on cerebral vessels (Knot and Nelson 1998; Osol et al. 2002; Cipolla et al. 2014a; Li and Brayden 2017). The intracellular Ca^2+^ level $\xi _0$ is directly associated with the concentration level of the Calcium-Calmodulin complex, which in turn promotes through MLCK the phosphorylation of myosin LC_20_. This cascade of events/processes is condensed into the Ca^2+^-dependent function ${\chi _5(\xi _0)}$, representing the normalized MLCK capacity to phosphorylate LC_20_. The fraction of phosphorylated LC_20_ (pLC_20_), normalized with respect to the maximum phosphorylatable fraction, is indicated with $\xi _5$, whilst $1-\xi _5$ represents the normalized fraction of non-phosphorylated LC_20_ that can be phosphorylated.

Also, the pressure-induced ROCK and PKC pathways involve a myriad of proteins and messengers, and here we represent them only through few intracellular variables. The ROCK activity level $\xi _1$ is up-regulated through pressure-dependent function ${\chi _1(\bar{P})}$ and is expected to impact the phosphorylation levels of the proteins MLCP (pMLCP) and Cofilin (pCofilin), indicated with $\xi _3$ and $\xi _4$, through the logistic functions ${\chi _3(\xi _1)}$ and ${\chi _4(\xi _1)}$. The phosphorylation of MLCP plays a pivotal role as Ca^2+^-sensitization mechanism as it reduces the MLCP’s capacity to de-phosphorylate LC_20_, which is described via ${\chi _6(\xi _3)}$. For describing the combined effect of MLCK and MLCP on LC_20_ we do not use the standard logic-based formalism as in Irons and Humphrey (2020). Since MLCK acts on the non-phosphorylated LC_20_ fraction, we can write the mass balance of phosphorylated LC_20_ fraction (coinciding with the ratio of activated myosin filaments over the total, $\xi _5$) as:

4 $$\begin{aligned} {\frac{{\text {d}} \xi _5}{{\text {d}}t}=\frac{1}{\tau _5}[ {\chi _5(\xi _0)}(1-\xi _5)-(1-{\chi _6(\xi _3)})\xi _5]}, \end{aligned}$$

where $\tau _5$ is the characteristic time constant for the dynamic regulating $\xi _5$. On the other hand, the phosphorylation of Cofilin impacts cytoskeleton remodelling by down-regulating the level of G-actin content/activity $\xi _6$ via the logistic function ${\chi _7(\xi _4)}$. Similarly, the pressure-activated PKC pathway is able to modify the cytoskeletal stiffness by promoting (through $\chi _2(\bar{P})$) heat shock protein 27 (HSP27) phosphorylation (pHSP27), indicated with the variable $\xi _2$, which in turn down-regulates $\xi _6$ via ${\chi _8(\xi _2)}$. To recap, the considered intracellular variables for this network are the normalized values (with respect to their maximum) of Ca^2+^ concentration ($\xi _0$), ROCK activity level ($\xi _1$), HSP27 phosphorylation level ($\xi _2$), MLCP phosphorylation level ($\xi _3$), Cofilin phosphorylation level ($\xi _4$), LC_20_ phosphorylation level ($\xi _5$), and G-actin content ($\xi _6$). The resulting system of ODEs is reported below:

5 $$\begin{aligned} \frac{{\text {d}} \xi _0}{{\text {d}}t}&= \frac{1}{\tau _0}({\chi _0(\bar{P})}-\xi _0), \end{aligned}$$

6 $$\begin{aligned} \frac{{\text {d}} \xi _1}{{\text {d}}t}&= \frac{1}{\tau _1}({\chi _1(\bar{P})}-\xi _1), \end{aligned}$$

7 $$\begin{aligned} \frac{{\text {d}} \xi _2}{{\text {d}}t}&= \frac{1}{\tau _2}({\chi _2(\bar{P})}-\xi _2), \end{aligned}$$

8 $$\begin{aligned} \frac{{\text {d}} \xi _3}{{\text {d}}t}&= \frac{1}{\tau _3}(\chi _3(\xi _1)-\xi _3), \end{aligned}$$

9 $$\begin{aligned} \frac{{\text {d}} \xi _4}{{\text {d}}t}&= \frac{1}{\tau _4}({\chi _4(\xi _1)}-\xi _4), \end{aligned}$$

10 $$\begin{aligned} \frac{{\text {d}} \xi _5}{{\text {d}}t}= & {} \frac{1}{\tau _5}[ {\chi _5(\xi _0)}(1-\xi _5)-(1-{\chi _6(\xi _3)})\xi _5], \end{aligned}$$

11 $$\begin{aligned} \frac{{\text {d}} \xi _6}{{\text {d}}t}&= \frac{1}{\tau _6}[(1-{\chi _7(\xi _4)})+(1-{\chi _8(\xi _2)})-(1-{\chi _7(\xi _4)})(1-{\chi _8(\xi _2)})-\xi _6]. \end{aligned}$$

Here, we assume that the myosin pLC_20_ coincides with the proportion of myosin filaments able to form cross-bridges with the actin filaments and produce power-stroke. The dimensional fraction of phosphorylated/activated myosin cross-bridges ($n_{\text {XB}}$) is retrieved as

12 $$\begin{aligned} n_{\text {XB}}=\xi _5 n_{\text {XBmax}}, \end{aligned}$$

where $n_{\text {XBmax}}$ is the maximum fraction of cross-bridges that can be activated (coinciding with the maximum myosin fraction that can be phosphorylated). The fraction $n_{\text {XB}}$, together with the level of actin polymerization (F-actin content, simply expressed as $\xi _7=1-\xi _6$) serve as input for the mechanical sub-model for tissue tone generation.

### Mechanical sub-model

The information from the signalling network drives the intracellular contractile machinery, which in turn is translated at tissue level through an active strain-energy function.

#### Contractile units and actin cortex

For computing tone generation, we follow one of the latest contributions by Murtada et al. (2016), based on previous filament-sliding models (Murtada et al. 2010, 2012) which have been experimentally validated by using SMCs from blood vessels. With respect to its predecessors, this model incorporates cytoskeleton contribution into the contractile machinery. Passive surrounding elements (mainly F-actin) constituting the actin cortex are connected with contractile units (CUs) arranged in series to form tissue contractile fibres (CFs). Here, the actin cortex stiffness ($k_{\text {AC}}$) is defined as a function of the F-actin content level ($\xi _7=1-\xi _6$)

13 $$\begin{aligned} k_{\text {AC}}=k_{\text {ACmax}}\frac{\xi _7^{n_{\text {AC}}}}{\xi _7^{n_{\text {AC}}}+{K_{\text {AC}}}^{n_{\text {AC}}}}, \end{aligned}$$

where $k_{\text {ACmax}}$ is the maximum stiffness under loading conditions, whilst $n_{\text {AC}}$ and $K_{\text {AC}}$ are the coefficients of the associated activation function. With respect to the work by Murtada et al. (2016), we assume that even if cross-bridges in the latched state exist, they do not contribute to the active tone generation. In this way, the total stiffness of all attached cross-bridges in half of a CU ($k_{\text {tCB}}$) can be simply evaluated as

14 $$\begin{aligned} k_{\text {tCB}}=\frac{L_\text {m}n_{\text {XB}}k_{\text {XB}}}{\delta _{\text {m}}}, \end{aligned}$$

where $L_\text {m}$ is the average length of myosin filaments, $\delta _{\text {m}}$ is the average distance between myosin monomers heads and $k_{\text {XB}}$ represents the elastic stiffness of the cross-bridge. As in Murtada et al. (2016) we define the total stiffness associated with a number of CUs ($N_{\text {CU}}$) as

15 $$\begin{aligned} k_{\text {tCU}}=\frac{\bar{L}_{\text {fo}}k_{\text {tCB}}}{2N_{\text {CU}}}, \end{aligned}$$

where $\bar{L}_{\text {fo}}$ is a normalized function representing the overlap between thin and thick filaments, to be introduced later on. The length of the CU in the deformed configuration ($l_{\text {SMC}}$) defined as

16 $$\begin{aligned} l_{\text {SMC}}=L_{\text {SMC}}+2N_{\text {CU}}(u_{\text {CB}} +u_{\text {fs}})+2u_{\text {FA}}, \end{aligned}$$

where $L_{\text {SMC}}$, $u_{\text {CB}}$, $u_{\text {fs}}$ and $u_{\text {FA}}$ are all quantities evaluated in the reference configuration. $L_{\text {SMC}}$ is the length of the CU, $u_{\text {CB}}$ is the average XB elongation, $u_{\text {fs}}$ is the relative filament sliding and $u_{\text {FA}}$ is the average surrounding passive elements elongation. From (16) the circumferential stretch $\lambda _{\theta }=l_{\text {SMC}}/L_{\text {SMC}}$ can be also defined as

17 $$\begin{aligned} \lambda _{\theta }=1+2N_{\text {CU}}(\bar{u}_{\text {CB}} +\bar{u}_{\text {fs}})+2\bar{u}_{\text {FA}}, \end{aligned}$$

where $\bar{u}_{\text {CB}}={u}_{\text {CB}}/L_{\text {SMC}}$, $\bar{u}_{\text {fs}}={u}_{\text {fs}}/L_{\text {SMC}}$ and $\bar{u}_{\text {FA}}=u_{\text {FA}}/L_{\text {SMC}}$. According to Murtada et al. (2016), the resulting reaction force due to the resistance from $N_{\text {CU}}$ in series with a F-actin element at each extremity is given as total displacement by the resulting stiffness of the system:

18 $$\begin{aligned} F_{\text {a}}=2(N_{\text {CU}}\bar{u}_{\text {CB}} +\bar{u}_{\text {FA}})\frac{k_{\text {tCU}}k_{\text {AC}}}{2k_{\text {tCU}} +k_{\text {AC}}}, \end{aligned}$$

whilst the average driving force generated from the cycling XBs results

19 $$\begin{aligned} F_{\text {c}}=\bar{L}_{\text {fo}} \frac{L_\text {m}}{\delta _\text {m}} n_{\text {XB}} k_{\text {XB}}u_{\text {PS}}, \end{aligned}$$

where $u_{\text {PS}}$ is the average displacement associated with power-stroke. The normalized filament overlap is described through a Gaussian function (Murtada et al. 2016) depending on the (normalized) relative filament sliding $\bar{u}_{\text {fs}}$:

20 $$\begin{aligned} \bar{L}_{\text {fo}}=\text {exp}\left[ \frac{1}{2}(\bar{u}_{\text {fs}} -\bar{u}_{\text {fs}}^{\text {opt}})^2/(s_{\text {f0}}/L_\text {m})^2\right], \end{aligned}$$

where $\bar{u}_{\text {fs}}^{\text {opt}}$ is the optimal filament sliding and $s_{\text {f0}}$ is a scaling parameter. A simple evolution law is used for describing the kinetics of the filament sliding caused by the XB cycling:

21 $$\begin{aligned} \frac{{\text {d}} \bar{u}_{\text {fs}}}{{\text {d}}t}=\beta _{\text {c}}(F_{\text {a}}-F_{\text {c}}), \end{aligned}$$

where $\beta _{\text {c}}$ is the time constant associated with the process. By considering (17), the active force $F_{\text {a}}$ can be re-written as a function of $\lambda _{\theta }$ and $\bar{u}_{\text {fs}}$:

22 $$\begin{aligned} F_{\text {a}}=(\lambda _{\theta }-1-2N_{\text {CU}}\bar{u}_{\text {fs}}) \frac{k_{\text {tCU}}k_{\text {AC}}}{2k_{\text {tCU}}+k_{\text {AC}}}. \end{aligned}$$

The generated tone from CFs can be quantified through the active First Piola-Kirchhoff stress $P_{\text {a}}$ of the tissue material, which can be evaluated as

23 $$\begin{aligned} P_{\text {a}}=N_{\text {CF}}F_{\text {a}}, \end{aligned}$$

where $N_{\text {CF}}$ is the surface density of the contractile fibres.

#### Vessel wall

The considered vascular tissue, consisting of different SMCs layers, is modelled as an axisymmetric homogeneous hyperelastic thick-walled tube. The luminal pressure *P* can be defined through the momentum conservation principle along the radial direction (Coccarelli et al. 2021)

24 $$\begin{aligned} P=P_{\text {ext}}+\int _{R_{\text {i}}}^{R_{\text {i}}+H} \left(\lambda _{\theta }\frac{\partial \Psi }{\partial \lambda _{\theta }}-\lambda _{r}\frac{\partial \Psi }{\partial \lambda _{r}}\right)\frac{dR}{\lambda _{\theta }\lambda _{z}r}, \end{aligned}$$

where $P_{\text {ext}}$ is the external pressure acting on the outer surface of the vessel (which in this study is assumed negligible), $\Psi$ is the material strain-energy function, $R_{\text {i}}$ and *H* are the inner radius and the thickness in the reference configuration $\Omega _{\text {R}}$, *r* is the radial coordinate in the deformed configuration $\Omega _{\text {D}}$, whilst $\lambda _{z}$ and $\lambda _{r}=\frac{R}{r k_{\omega }\lambda _{z}}$ are the axial and radial stretches, respectively, with $k_{\omega }$ accounting for the residual circumferential strain. Moreover, we assume that there is no difference in thickness between the load-free and reference configurations and the material is incompressible. This latter constraint allows the following mapping

25 $$\begin{aligned} \Omega _{\text {R}}\rightarrow \Omega _{\text {D}}:~~~r=\sqrt{\frac{(R_i+H)^2-R_i^2}{k_{\omega }\lambda _z}+r_i^2}, \end{aligned}$$

where $r_i$ is the deformed luminal radius. As usual, strain-energy function is decomposed into a passive and active components ($\Psi =\Psi _\text {p}+\Psi _\text {a}$), with the former defined as (Gasser et al. 2006)

26 $$\begin{aligned} \Psi _\text {p}=c_0(I_1-3)+\frac{c_1}{2c_2}\{\text {exp}[c_2(I_4-1)^2]-1\}, \end{aligned}$$

where $c_0$, $c_1$ and $c_2$ are the media constitutive parameters, $I_1=\lambda _{r}^2+\lambda _{\theta }^2+\lambda _{z}^2$, $I_4=\lambda _{\theta }^2 \text {cos}^2\phi +\lambda _{z}^2\text {sin}^2\phi$ with $\phi$ being the orientation angle (with respect to the circumferential direction) of a collagen fibres family. In line with Murtada and Holzapfel (2014), we assume that these collagen fibres are aligned with the contractile fibres CFs, which are oriented along the circumferential direction of the vessel ($\phi =0 ^{\circ }$). By assuming that collagen fibres cannot sustain compression loads, the anisotropic contribution ($I_4$ - 1) is set to zero when $I_4<$ 1. On the other hand, the strain-energy function component representing active tone generation in the SMC tissue can be obtained as in Murtada et al. (2010); Murtada and Holzapfel (2014)

27 $$\begin{aligned} \Psi _\text {a}=\int {P_\text {a}~d\lambda _{\theta }}=\frac{N_{\text {CF}}}{2} \frac{k_{\text {tCU}}k_{\text {AC}}}{2k_{\text {tCU}}+k_{\text {AC}}} (\lambda _{\theta }-1-2N_{\text {CU}}\bar{u}_{\text {fs}})^2. \end{aligned}$$

### Model parameter identification and solution procedure

The parameters of the chemical and mechanical sub-models were separately identified through a two-tiered approach by solving two optimization problems, which were defined in line with Coccarelli and Pant (2023). For the mechanical sub-model optimization problem, the best-fit parameters of the chemical sub-model were used. For each optimization problem, the solution is the set of model parameters that minimizes the discrepancy (through a cost function) between the simulated data and the corresponding measurements across a vessel population. The cost function is made of two components ($\mathcal {L}_\textrm{tot}$=$\mathcal {L}_{data}$+$\mathcal {L}_{\text {con}}^{sum}$), with $\mathcal {L}_{data}$ is the L2 norm of the simulated data with respect to average values from experiments, while $\mathcal {L}_{\text {con}}^{sum}$ is a penalty-based term accounting for the model constraints that variables/parameters must satisfy. These are introduced to refine and accelerate the search by discarding solutions which do not lie within the expected physical value ranges. Each constraint contribution is included into the cost function as an individual penalty $\mathcal {L}_{\text {con}}^{i}=\kappa \Delta y_{\text {con}}$, where $\kappa$ is the penalty parameter (1.0e3) and $\Delta y_{\text {con}}$ defines the absolute distance of the variable with respect to the allowed interval boundary. The parameter set is identified by minimizing the cost function through the covariance matrix adaptation evolution strategy (CMA-ES) (Hansen et al. 2009; Hansen 2016). In the CMA-ES settings, the maximum number of iterations ‘maxiter’ per search is set equal to 1.0e3, with a standard deviation ‘sigma0’ not exceeding 1. Due to the stochastic nature of the search algorithm (and the high number of model parameters), multiple runs were carried out to find best model fits.

In this study, we focus on the steady-state behaviour of the model. Hence, the associated time constants ($\tau _0, \tau _1, \tau _2, \tau _3, \tau _4, \tau _5, \tau _6$ and $\beta _{\text {c}}$) are all set equal to 1. For any pressure level, the static solution of the chemical sub-model nodal variables was obtained by solving the system of ODEs (5)–(11). Regarding the mechanical sub-model, we only consider the $\bar{u}_{\text {fs}}$ evaluated with a circumferential stretch averaged over the vessel thickness, which is representative of the mean filament sliding occurring across the wall. Furthermore, (24) is re-arranged as a function of the luminal radius in the deformed configuration $r_i=r_i(P, \bar{u}_{\text {fs}})$, which can be internally evaluated within the solution procedure for computing $\frac{{\text {d}}\bar{u}_{\text {fs}}}{{\text {d}}t}=\frac{{\text {d}}\bar{u}_{\text {fs}}}{{\text {d}}t}(\bar{u}_{\text {fs}}, r_i(P, \bar{u}_{\text {fs}}))$ via (21). Hence, the latter equation is solved at steady-state for obtaining $r_i$ and $\bar{u}_{\text {fs}}$ at each pressure level. For the integral in (24) Simpson’s rule is used. Both sub-models were implemented in Python3.9 and all equations were solved by means of the function ‘root’ (method =‘lm’) from the SciPy library (version 1.6.0), while for the optimizer we used the function ‘fmin2’ from the cma library (version 3.2.2).

## Results

### Cell signalling sub-model

Here, we report how the chemical sub-model is able to capture the experimental data by Johnson et al. (2009), El-Yazbi et al. (2010), Moreno-Dominguez et al. (2014) obtained by employing pressure myography on middle cerebral arteries from Sprague Dawley rats. These studies report the levels of pHSP27, pMLCP, pCofilin, pLC_20_ and G-actin for different pressures (ranging from 10 to 140 mmHg), under control and pharmacologically manipulated conditions. For this purpose the selective ROCK inhibitor H-1152 dihydrochloride (H1152) and PKC inhibitor GF109203X (GF) were used. For the signalling network variables normalization, we considered a maximum pressure $P_{\text {max}}$ equal to 150 mmHg, beyond which all the considered intra-cellular processes reach saturation level. H1152 doses of 0.3 $\mu$M and 0.5 $\mu$M are expected to inhibit the ROCK pathway and were simulated by setting the network variable $\xi _1$ equal to 0.12 and 0.07, respectively (in line with the H1152 dose-inhibition curve reported in Johnson et al. (2009)). On the other hand, 3.0 $\mu$M GF is expected to block the PKC pathway, and therefore, this condition was simulated by setting the network variable $\xi _2$ equal to 0.1. The experimental data used for the model parameters identification are summarized in the Appendix.

Regarding the optimization procedure constraints, we ensured that the pressure-cytosolic Ca^2+^ curve (${\chi _0(\bar{P})}$) lies within the maximum ranges observed among the experimental data reported in Knot and Nelson (1998), Osol et al. (2002), Cipolla et al. (2014a), Li and Brayden (2017) (see Appendix). These experimentally obtained curves exhibit different features but for each of them the EC_50_ appears to be significantly higher than 15 mmHg. Here to refine and accelerate the search we add the constraint which penalizes parameters sets providing an EC_50_ for ${\chi _0(\bar{P})}$ that is lower than 0.1. We also impose that, in case of extracellular Ca^2+^ removal (simulated by setting the network variable $\xi _0$ equal to 0.1), the pLC_20_ cannot exceed the value in the control case at 10 mmHg, for pressure increasing up to 120 mmHg. In line with the recordings by Johnson et al. (2009), $n_{\text {XBmax}}$ was assumed to be 0.55 (this is necessary for passing the information from the chemical sub-model to the mechanical one).

These settings enabled us to identify the coefficients ($B_0$, *K* and *n*) of the nine logistic functions (composing the signalling network) which best capture the above-mentioned experimental data (Johnson et al. 2009; El-Yazbi et al. 2010; Moreno-Dominguez et al. 2014) (see Table 1).

**Table 1:** Coefficients of the logistic functions obtained through chemical model fitting against experimental recordings (Johnson et al. 2009; El-Yazbi et al. 2010; Moreno-Dominguez et al. 2014)

	${\chi _0(\bar{P})}$	${\chi _1(\bar{P})}$	${\chi _2(\bar{P})}$	${\chi _3(\xi _1)}$	${\chi _4(\xi _1)}$	${\chi _5(\xi _0)}$	${\chi _6(\xi _3)}$	${\chi _7(\xi _4)}$	${\chi _8(\xi _2)}$
$B_0$	0.131	0.181	0.000	0.000	0.419	0.000	0.020	0.187	0.142
*K*	15.470	6413.033	0.926	0.512	0.219	0.423	0.332	0.440	4401.162
*n*	0.464	3.083	0.558	1.325	15.825	521.545	10.680	88.701	1.408

**Fig. 2:**
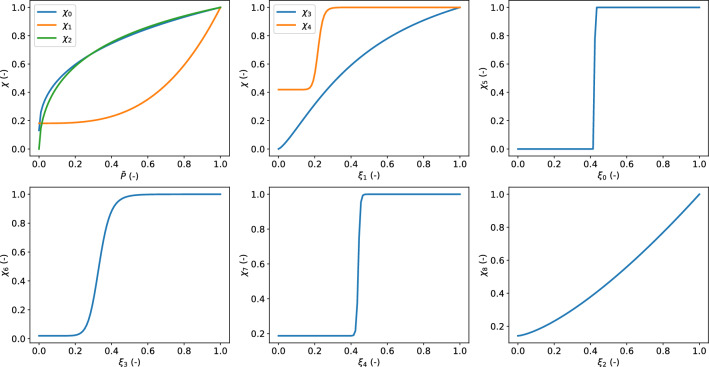
Logistic functions of the signalling network depicted in Fig. 1. The associated coefficients (obtained through optimization of the chemical sub-model parameters) are reported in Table 1

**Fig. 3:**
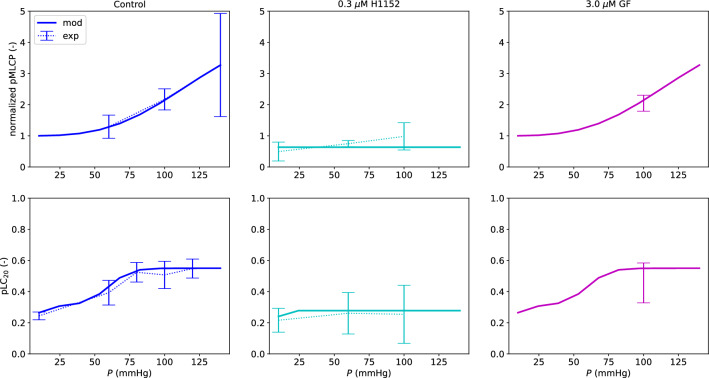
Chemical model fitting - pMLCP and pLC_20_. The experimental data are reported as mean value ± standard deviation. The reported pMLCP values are normalized with respect to their control case at 10 mmHg. The experimental measurements of pMLCP and pLC_20_ under 3.0 $\mu$M GF in Johnson et al. (2009) were reported with respect to the 100 mmHg control case. Here, these values were re-scaled with respect to the reference control values. The standard deviations were re-calculated accordingly (by assuming that the quantities are independent variables)

The logistic functions constituting the cell signalling network are reported in Fig. 2. Figure 3 shows how the cell signalling sub-model is able to capture the influence of pressure on the pMLCP and pLC_20_ levels in the control case, under 0.3 $\mu$M H1152 and 3.0 $\mu$M GF. While for pMLCP values are normalized with respect to the 10 mmHg control case, absolute values are reported for pLC_20_ fractions. The experimental pLC_20_ value under 3.0 $\mu$M GF was re-scaled (multiplied by a factor $\approx 1.1$) by considering the difference between the two control values at 100 mmHg reported in Johnson et al. (2009) (Figs. 8B and 10E therein).

**Fig. 4:**
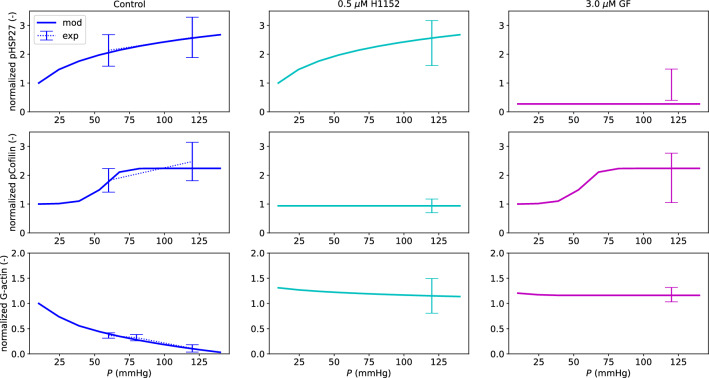
Chemical model fitting - pHSP27, pCofilin and G-actin. The experimental data are reported as mean value ± standard deviation. All the reported values are normalized with respect to their control case at 10 mmHg. In the 3.0 $\mu$M GF case, pHSP27 ($\xi _2$) was set equal to 0.1 across the whole pressure range to simulate the drug effect

The effect of pressure on the intracellular pathways governing the cytoskeleton remodelling is reported in Fig. 4. The defined chemical sub-model is able to reproduce the pressure-induced pHSP27 elevation in the control case and under 0.5 $\mu$M H1152. Similarly, the relationships between pressure and pCofilin, and pressure and G-actin levels are well captured in the control case, under 0.5 $\mu$M H1152 and 3.0 $\mu$M GF.

### Vascular tissue sub-model

Here, we considered other vessels data from Johnson et al. (2009) to fit the mechanical sub-model and validate the global methodology. The simulated protocol consists of tube inflation tests at different pressure levels under fixed axial length, with the addition/removal of vasoactive substances. A first group of five vessels (here renamed ‘vessels group 1’) was used for comparing the H1152 effect on diameter with respect to the control and extracellular Ca^2+^ removal cases. Similarly, GF effect was tested on another group of six arteries (here referred as ‘vessels group 2’). From the reported experimental data, the only main difference between these two vessels groups seems to be the average size, with the group 1 having a slighter smaller radius near to the load-free condition ($R_o'$=0.011 cm for vessels group 1, $R_o'$=0.0126 cm for vessels group 2). For the vessels used in Johnson et al. (2009), the load-free thickness-medium radius ratio ($h_w$), the axial stretch ($\lambda _z$) and the circumferential pre-stretch ($k_{\omega }$) were not reported. We used the pressure-diameter data derived from the assessment on vessels group 1 for identifying (through the methodology reported in Sect. 2.3) these geometric factors alongside some passive and active response parameters ($c_0$, $c_2$, $u_{\text {PS}}$, $\frac{k_{\text {XB}}}{\delta _{\text {m}}}$, $\bar{u}_{\text {fs}}^{\text {opt}}$, $s_{\text {fo}}$, $k_{\text {ACmax}}$, $n_{\text {AC}}$, $K_{\text {AC}}$). Other model parameters are expected to vary less among SMCs, and therefore, they were assumed equal to what is reported in the literature (Murtada and Holzapfel 2014; Murtada et al. 2016). The chemical sub-model parameters (as well as the way in which drug interventions are reproduced) are the same as in Sect. 3.1. Extracellular Ca^2+^ removal dramatically reduces the intracellular Ca^2+^ level due to Ca^2+^ influx abolition. This condition is modelled by setting the network node $\xi _0$ equal to 0.1. To accelerate and refine the search, we penalized (as reported in Sect. 2.3) any set of parameters for which i) $|\bar{u}_{\text {fs}}|$ exceeds 0.1 and/or ii) the derivative of the outer diameter (in the deformed configuration) $D_o$ with respect to *P* under control conditions is positive across the range 60–100 mmHg. All the parameters of the mechanical sub-model (including the ones obtained though fitting against the experimental curves) are summarized in Table 2.

**Table 2:** Mechanical model parameters. It is noticed that in the current model formulation only the ratio (and not the absolute values) between $k_{\text {XB}}$ and $\delta _{\text {m}}$ is required. Some of the fitted parameters were restrained between specific ranges of values: 0.13 $\le$ $h_w$ $\le$ 0.40, 1.0 $\le$ $\lambda _z$ $\le$ 1.5, 1.0 $\le$ $k_{\omega }$ $\le$ 1.5, 0.1 r.v. $\le$ $u_{\text {PS}}$ $\le$ 10.0 r.v., 0.1 r.v. $\le$ $\frac{k_{\text {XB}}}{\delta _{\text {m}}}$ $\le$ 10.0 r.v., 0.1 r.v. $\le$ $k_{\text {ACmax}}$ $\le$ 10.0 r.v. where r.v. is the reference value from Murtada et al. (2016)

Parameter	Description	Value
*Geometry*		
$R_o'$ (cm)	Load-free outer radius	0.0110, 0.0126 - assumed
$h_w$ (-)	Load-free thickness-medium radius ratio	0.367 - fitted
$\lambda _z$ (-)	Axial stretch	1.364 - fitted
$k_{\omega }$ (-)	Circumferential pre-stretch	1.373 - fitted
*Passive response*		
$c_0$ (dyne cm^-2^)	Material constant	9.1633 · 10^4^ - fitted
$c_1$ (dyne cm^-2^)	Material constant	3.15 · 10^4^ - from Murtada and Holzapfel (2014)
$c_2$ (-)	Material constant	0.646 - fitted
$\phi$ (^∘^)	Fibres orientation angle	0.0 - assumed
*Active response*		
$N_{\text {CF}}$ (cm^-2^)	Number of CF per unit area	2.44 · 10^10^ - from Murtada et al. (2016)
$L_{\text {SMC}}$ (cm)	SMC length	0.01 - from Murtada et al. (2016)
$L_{\text {m}}$ (cm)	Myosin filament length	3.0 · 10^-5^ - from Murtada et al. (2016)
$u_{\text {PS}}$ (cm)	Average elongation caused by power-stroke	1.424 · 10^-6^ - fitted
$\frac{k_{\text {XB}}}{\delta _{\text {m}}}$ (dyne cm^-2^)	Cross-bridge elastic stiffness per distance between myosin monomers	8.166777 · 10^6^ - fitted
$\bar{u}_{\text {fs}}^{\text {opt}(-)}$(-)	Normalized optimal filament sliding	1.490 · 10^-2^ - fitted
$s_{\text {fo}}$ (cm)	Filament overlap scaling factor	5.870 · 10^-7^ - fitted
$k_{\text {ACmax}}$ (dyne cm^-1^)	Actin cortex stiffness scaling factor	1.004 · 10^1^ - fitted
$n_{\text {AC}}$ (-)	Actin cortex activation function coefficient	4.156 - fitted
$K_{\text {AC}}$ (-)	Actin cortex activation function coefficient	10.201 - fitted

It is worth noticing that with this set of parameters the system reaches steady-state conditions for all the considered pressure levels (see Appendix). Geometric factors such as axial stretch ($\lambda _z$) and circumferential pre-stretch ($k_{\omega }$) may vary quite a lot from vessel to vessel in the brain and depend of course on the experimental procedure (Monson et al. 2008). Furthermore, for resistance cerebral vessels, the role of axial stretch on their pressure-induced vasoreactivity is not fully clarified (Coats and Hillier 1999). The identified parameters need therefore to be interpreted as representative of the mean behaviour of the vessels group.

**Fig. 5:**
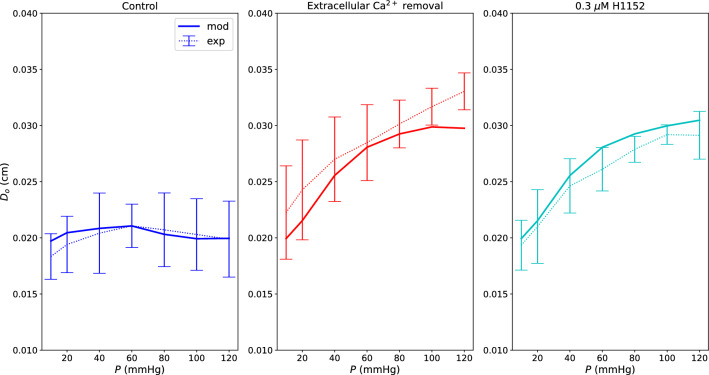
Mechanical model fitting for vessels group 1 under control, extracellular Ca^2+^ removal and 0.3 $\mu$M H1152 conditions. $D_o$ is the outer diameter in the deformed configuration. The experimental data are reported as mean value ± standard deviation

**Fig. 6:**
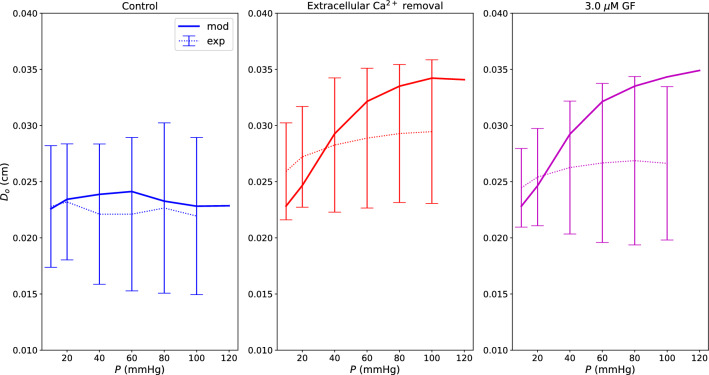
Mechanical model validation for vessels group 2 under control, extracellular Ca^2+^ removal and 3.0 $\mu$M GF conditions. $D_o$ is the outer diameter in the deformed configuration. The experimental data are reported as mean value ± standard deviation

Figure 5 shows how the model captures the pressure-induced tone under control, extracellular Ca^2+^ removal and H1152 inhibition conditions. In the control case, vessels are able to limit variations in their diameter despite pressure elevation. On the other hand, by removing extracellular Ca^2+^, we obtain a situation very close to the pure passive vessel behaviour. The H1152 case shows the impact of ROCK inhibition on the SMC contractile machinery. In the latter case tone generation is compromised similarly to the case of extracellular Ca^2+^ absence. Then, we used the pressure-outer diameter data concerning the vessels group 2 for validating the chemo-mechanical model. This group of vessels appeared to be slightly bigger (different load-free radius) and was subjected to 3.0 $\mu$M GF (simulated as in Sect. 3.1). Overall, for these vessels, predictions fall within the experimentally observed ranges (Fig. 6). It is important to notice that these arteries may have different geometric and passive material properties with respect to the vessel group 1. Furthermore, we assumed that GF impacts the contractile machinery only through the PKC pathway, without considering potential secondary effects on the remaining signalling network. These may be some of the reasons for the observed discrepancies between simulated behaviour and experimental mean values in the pharmacologically manipulated cases.

**Fig. 7:**
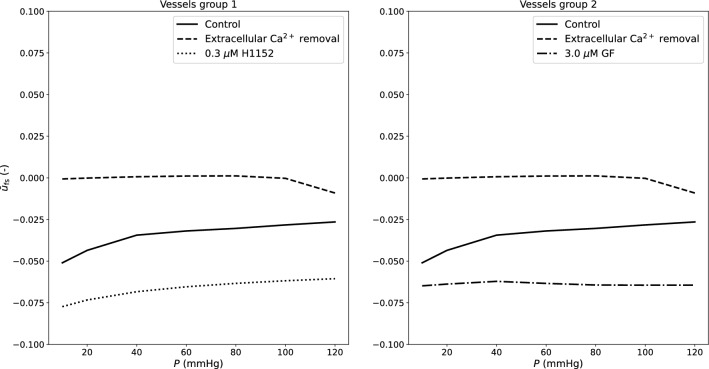
Simulation results - role of luminal pressure on normalized relative filament sliding for vessels group 1 and group 2

In Fig. 7, we show how the (normalized) relative filament sliding changes upon pressure elevation across all the considered conditions and vessels groups. Interestingly, in the control cases, the sliding between filaments is limited (slight contraction) across the whole considered pressure range. Extracellular Ca^2+^ removal hinders the formation of new phosphorylated XBs, leaving the vessel to expand upon pressure loading. On the contrary, alteration of the cytoskeleton function through drugs seems to have the opposite effect (filaments highly contracted).

**Fig. 8:**
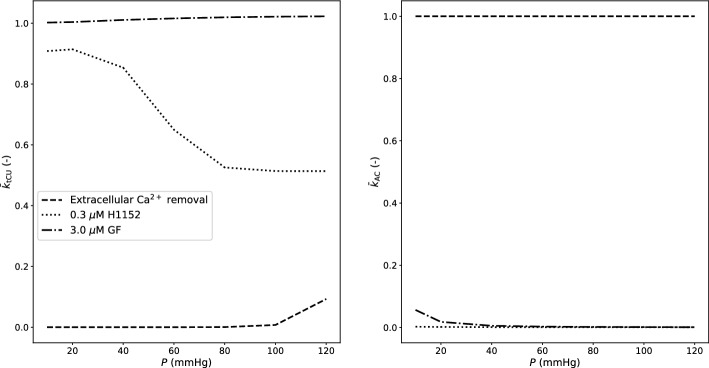
Simulation results—role of luminal pressure on total CU stiffness and actin cortex stiffness. $\bar{k}_{\text {tCU}}$ is the total CU stiffness normalized with respect to the corresponding control value. $\bar{k}_{\text {AC}}$ is the actin cortex stiffness normalized with respect to the corresponding control value

Pressure-induced signalling pathways ultimately alter the stiffness of contractile units and actin-cortex (Fig. 8). The extracellular Ca^2+^ removal case, as expected, has a profound limiting effect on pressure-induced CU stiffness, while the actin cortex behaviour coincides with the control one. Both drug interventions almost nullify the stiffness of the actin cortex across the considered pressure range. On the other hand, while inhibition of the ROCK pathway limits pressure-induced CU stiffness, the GF case exhibits a stiffness increase with pressure that is even slightly higher than the control one. This may be due to the fact that CU stiffness also depends on $\bar{u}_{\text {fs}}$, which is significantly different between the GF and the control case. Although some of the reported numerical findings may appear intuitive, these still need to be corroborated by future experimental studies.

## Discussion and concluding remarks

Through this work we introduced a new multi-scale model for computing pressure-induced tone and resulting diameter in SCAs. Luminal pressure is converted into vessel tone through a framework made of a signalling network, accounting for the intracellular Ca^2+^, ROCK and PKC pathways, which feeds a mechanical sub-model combining actin–myosin interaction with cytoskeleton remodelling to evaluate tissue contractility. These sub-models were fitted and validated against experimental data by considering control conditions and with selective intracellular pathway manipulations. The model was also used for assessing how stiffness of the contractile machinery components vary with pressure and with/without pharmacological intervention. So far, the vast majority of vascular SMC contractility models (Yang et al. 2003a; Murtada et al. 2010; Carlson and Beard 2011; Murtada et al. 2012; Edwards and Layton 2014; Murtada et al. 2016; Coccarelli et al. 2018) describe the XB kinetics by using the so-called ‘Hai and Murphy model’ (Hai and Murphy 1988), which hypothesizes a contribution to force generation by XBs in a latched state. Through this study, we showed that this four-state kinetic model is not strictly necessary for reproducing the behaviours observed in experiments, as long as MLCP and cytoskeleton regulation are accounted for. Upon a more detailed experimental characterization of the XB dynamics, complex models such as (Hai and Murphy 1988) could also be integrated in the proposed framework.

The main limitations of the study are intrinsically associated with the uncertainties regarding different cellular processes and cell-tissue signal transduction. Some alleged secondary signalling connections between cellular components which so far have been only partially explored may play a role in pressure-induced contractility and therefore require further elucidation. An earlier study (Gokina et al. 2002) reported indeed that, at intermediate pressure levels, drugs which prevent/inhibit actin polymerization had also an effect on the cellular Ca^2+^ influx by modifying the activity of voltage gated Ca^2+^ channels. Mufti et al. (2010) proposed that Ca^2+^ waves originated from sarco-plasmic reticulum can also modulate MLCP activity. However, the lack of further experimental evidence together with the scarce mention by reference works (Cole and Welsh 2011; Walsh and William 2013) made us consider these connections of secondary importance (and therefore omitted). Ca^2+^ dynamics is pivotal for the initiation of tone generation (Jackson 2021) and certainly its modelling representation can be enhanced. A more complete experimental characterization of these processes will certainly help us to model with higher accuracy selective interventions targeting cell contractility (such as GF). Furthermore, we mention that the proposed model does not include yet a detailed link between integrins and Ca^2+^ sensitization processes (Colinas et al. 2015), which could be extremely useful in the future for simulating drugs that impact the signalling between SMC and extra-cellular matrix.

Given the high number of model parameters to be identified, we conducted numerous CMA-ES runs, which yielded multiple solutions in the parameter space. Among these, we identified the set of parameters which minimizes the associated cost function and that is most likely close to the global minima of the optimization problem. While a systematic identifiability study can be performed in the future, additional experimental data may be necessary to guarantee well-posedness of the inverse problem and uniqueness of parameters. The chemical sub-model was developed by considering only the data from one laboratory (Johnson et al. 2009; El-Yazbi et al. 2010; Moreno-Dominguez et al. 2014) and therefore a broader validation by using new data from different research groups will be necessary for reinforcing its predictive power and robustness. On the other hand, the mechanical sub-model of our framework is based on the very established work by Murtada et al. (2010, 2012, 2016) and, in the future, it could be further enhanced by using displacement-active tension recordings from wire myograph settings. All the presented results were at steady-state conditions, but we are currently investigating how the model is able to reproduce and predict time-dependent behaviours.

Being one of the earliest attempts to quantify the major known contributors to vascular SMC contractile machinery in SCAs, the proposed minimalistic modelling approach aims to provide reliable predictions without sacrificing its interpretability. However, as per the methodology proposed in Irons and Humphrey (2020), Irons et al. (2021), new components can be easily incorporated into the framework once new experimental data are made available. In addition, differences in pressure-induced vasoreactivity between cerebral resistance arteries and arterioles seem mainly due to different Ca^2+^ activity rather than Ca^2+^ sensitivity (Cipolla et al. 2014a). Hence, upon adequate characterization of the pressure-Ca^2+^ relationship, the current model could be also used for modelling the behaviour of small cerebral vessels. Finally, since alterations in some of the considered cellular pathways (such as ROCK) are associated with different vascular pathological conditions (Chrissobolis and Sobey 2001; El-Yazbi and Abd-Elrahman 2017), the proposed methodology may also constitute an in silico basis for the design and testing of novel therapies.

## Figures and Tables

**Fig. 9:**
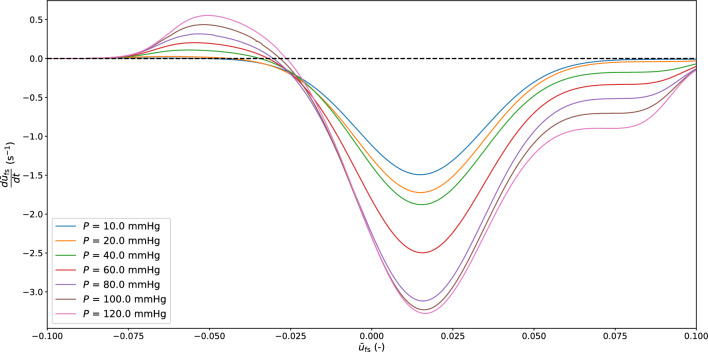
Dependency of $\frac{{\text {d}}\bar{u}_{\text {fs}}}{{\text {d}}t}$ on $\bar{u}_{\text {fs}}$ across the pressure range 10–120 mmHg. For each pressure level, intersection of the associated curve with the horizontal dashed line indicates a steady-state solution

## Data Availability

The simulation data and computer code are available from the corresponding author, A.C., upon reasonable request.

## Appendix

### Experimental data for chemical sub-model parameters identification

Experimental data for the identification of the chemical sub-model parameters are reported in Table 3 (control conditions), Table 4 (H1152 stimulation) and Table 5 (GF stimulation).

**Table 3:** Experimentally recorded mean values of normalized (with respect to 10 mmHg control case, indicated with superscript ‘0’) pHSP27, pMLCP, pCofilin, pLC_20_ and G-actin under control conditions used for model fitting

*P* (mmHg)	$\xi _2/\xi _2^0$ (–)	$\xi _3/\xi _3^0$ (–)	$\xi _4/\xi _4^0$ (–)	$\xi _5/\xi _5^0$(–)	$\xi _6/\xi _6^0$ (–)
60	2.134 (Moreno-Dominguez et al. 2014	1.292 (Johnson et al. 2009)	1.821 (Moreno-Dominguez et al. 2014)	1.611 (Johnson et al. 2009)	0.365 (Moreno-Dominguez et al. 2014)
80	–	–	–	2.148 (Moreno-Dominguez et al. 2014	0.322 (Moreno-Dominguez et al. 2014)
100	–	2.169 (Johnson et al. 2009)	–	2.078 (Johnson et al. 2009)	–
120	2.582 (Moreno-Dominguez et al. 2014)	–	2.476 (Moreno-Dominguez et al. 2014)	2.246 (Moreno-Dominguez et al. 2014)	0.110 (Moreno-Dominguez et al. 2014)
140	–	3.272 (El-Yazbi et al. 2010)	–	–	–

**Table 4:** Experimentally recorded mean values of normalized (with respect to 10 mmHg control case, indicated with superscript ‘0’) pHSP27, pMLCP, pCofilin, pLC_20_ and G-actin under 0.3 $\mu$M H1152 inhibition used for model fitting

*P* (mmHg)	$\xi _2/\xi _2^0$ (–)	$\xi _3/\xi _3^0$ (–)	$\xi _4/\xi _4^0$ (–)	$\xi _5/\xi _5^0$(–)	$\xi _6/\xi _6^0$ (–)
60	–	0.494 (Johnson et al. 2009)	–	1.208 (Johnson et al. 2009)	–
100	–	0.981 (Johnson et al. 2009)	–	1.176 (Johnson et al. 2009)	–
120	2.387 (Moreno-Dominguez et al. 2014)	–	0.938 (Moreno-Dominguez et al. 2014)	–	1.151 (Moreno-Dominguez et al. 2014)

**Table 5:** Experimentally recorded mean values of normalized (with respect to 10 mmHg control case, indicated with superscript ‘0’) pHSP27, pMLCP, pCofilin, pLC_20_ and G-actin under 3.0 $\mu$M GF inhibition used for model fitting. *Obtained by re-normalizing the original experimental data with respect to $\xi _2@$10 mmHg control case. **obtained by re-scaling the original experimental data with respect to the reference $\xi _5@$100 mmHg control case (scaling factor $\approx$ 1.1)

*P* (mmHg)	$\xi _2/\xi _2^0$ (–)	$\xi _3/\xi _3^0$ (–)	$\xi _4/\xi _4^0$ (–)	$\xi _5/\xi _5^0$(–)	$\xi _6/\xi _6^0$ (–)
100	–	2.045^∗^ (Johnson et al. 2009)	–	0.456^∗∗^ (Johnson et al. 2009)	–
120	0.942 (Moreno-Dominguez et al. 2014)	–	1.907 (Moreno-Dominguez et al. 2014)	–	1.175 (Moreno-Dominguez et al. 2014)

### Boundaries of pressure-Ca^2+^ relationship

For the chemical sub-model parameter identification, we imposed that the curve representing pressure-Ca^2+^ relationship (${\chi _0(\bar{P})}$) must be confined within some defined boundaries. Here, we considered the experimentally recorded traces from different works (Knot and Nelson 1998; Osol et al. 2002; Cipolla et al. 2014a; Li and Brayden 2017) and we normalized them with respect to their value at 10 mmHg. Among these, the experimentally derived curve by Osol et al. (2002) had the highest values across the considered pressure range. For our normalized pressure-Ca^2+^ function, we defined as upper boundary the data (mean value plus standard deviation) from the latter study (Table 6).

**Table 6:** Experimental data from Osol et al. (2002). Ca^2+^ data, reported as mean value ± standard deviation, was normalized with respect to the value at 10 mmHg

*P* (mmHg)	Ca^2+^ measurement (–)	Ca^2+^ boundary (–)
20	1.092 ± 0.693	1.785
30	1.215 ± 0.959	2.175
40	1.554 ± 1.332	2.886
50	2.061 ± 1.386	3.447
60	2.938 ± 0.586	3.525
100	3.308 ± 0.640	3.947
140	3.619 ± 0.679	4.298

On the other hand, since pressure causes an increase in cytosolic Ca^2+^, we imposed that across the considered pressure range the normalized pressure-Ca^2+^ function cannot go below 1.0.

### Mechanical sub-model steady-states

In Fig. 9 the dependency of $\frac{{\text {d}}\bar{u}_{\text {fs}}}{{\text {d}}t}$ on $\bar{u}_{\text {fs}}$ for different pressure levels is illustrated.

## References

[CR1] Carlson B, Beard D (2011). Mechanical control of cation channels in the myogenic response. Am J Physiol Heart Circ Physiol.

[CR2] Chrissobolis S, Sobey C (2001). Evidence that rho-kinase activity contributes to cerebral vascular tone in vivo and is enhanced during chronic hypertension. Circ Res.

[CR3] Cipolla M et al (2014a) Increased pressure-induced tone in rat parenchymal arterioles vs. middle cerebral arteries: role of ion channels and calcium sensitivity. J Appl Physiol 117:53–59 10.1152/japplphysiol.00253.2014 PMC4101583 24790017

[CR4] Cipolla M et al (2014b) Postischemic reperfusion causes smooth muscle calcium sensitization and vasoconstriction of parenchymal arterioles. Stroke 45:2425–2430 10.1161/STROKEAHA.114.005888 PMC4144269 24968928

[CR5] Cipolla M, Gokina N, Osol G (2002). Pressure-induced actin polymerization in vascular smooth muscle as a mechanism underlying myogenic behavior. FASEB J.

[CR6] Coats P, Hillier C (1999). Determination of an optimal axial-length tension for the study of isolated resistance arteries on a pressure myograph. Exp Physiol.

[CR7] Coccarelli A et al (2021) A framework for incorporating 3D hyperelastic vascular wall models in 1d blood flow simulations. Biomech Model Mechanobiol 20:1231–1249 10.1007/s10237-021-01437-5 PMC8298378 33683514

[CR8] Coccarelli A, Pant S (2023) On the Ca^2+^ elevation in vascular endothelial cells due to inositol trisphosphate-sensitive store receptor activation: A data-driven modeling approach. Comput Biol Med 164:107111 10.1016/j.compbiomed.2023.107111 37540925

[CR9] Coccarelli A, Edwards D, Aggarwal A, Nithiarasu P, Parthimos D (2018). A multiscale active structural model of the arterial wall accounting for smooth muscle dynamics. J R Soc Interface.

[CR10] Cole W, Welsh D (2011). Role of myosin light chain kinase and myosin light chain phosphatase in the resistance arterial myogenic response to intravascular pressure. Arch Biochem Biophys.

[CR11] Colinas O (2015). $$\alpha $$5-integrin-mediated cellular signaling contributes to the myogenic response of cerebral resistance arteries. Biochem Pharmacol.

[CR12] Cui Y et al (2022) Myogenic vasoconstriction requires canonical Gq/11 signaling of the Angiotensin II Type 1 Receptor. J Am Heart Assoc 11(4):e022070 10.1161/JAHA.121.022070 PMC9245832 35132870

[CR13] Davis M (2012). Perspective: physiological role(s) of the vascular myogenic response. Microcirculation.

[CR14] Edwards A, Layton A (2014). Calcium dynamics underlying the myogenic response of the renal afferent arteriole. Am J Physiol Renal Physiol.

[CR15] El-Yazbi A (2010). Pressure-dependent contribution of rho kinase-mediated calcium sensitization in serotonin-evoked vasoconstriction of rat cerebral arteries. J Physiol.

[CR16] El-Yazbi A, Abd-Elrahman K (2017) ROK and arteriolar myogenic tone generation: molecular evidence in health and disease. Front Pharmacol 8:87 10.3389/fphar.2017.00087 PMC5322222 28280468

[CR17] El-Yazbi A, Abd-Elrahman K, Moreno-Dominguez A (2015) PKC-mediated cerebral vasoconstriction: role of myosin light chain phosphorylation versus actin cytoskeleton reorganization. Biochem Pharmacol 95:263–278 10.1016/j.bcp.2015.04.011 25931148

[CR18] Fan J-L (2022). Integrative cerebral blood flow regulation in ischemic stroke. J Cereb Blood Flow Metab.

[CR19] Gasser TC, Ogden RY, Holzapfel GA (2006). Hyperelastic modelling of arterial layers with distributed collagen fibre orientations. J R Soc Interface.

[CR20] Gokina N, Osol G (2002) Actin cytoskeletal modulation of pressure-induced depolarization and Ca^2+^ influx in cerebral arteries. Am J Physiol Heart Circ Physiol 282:H1410–H1420 10.1152/ajpheart.00441.2001 11893578

[CR21] Gokina N et al (2005) Effects of Rho kinase inhibition on cerebral artery myogenic tone and reactivity. J Appl Physiol 98:1940–1948 10.1152/japplphysiol.01104.2004 15626753

[CR22] Good M et al (2018) Endothelial cell Pannexin1 modulates severity of ischemic stroke by regulating cerebral inflammation and myogenic tone. JCI Insight 3(6):e96272 10.1172/jci.insight.96272 PMC5926909 29563335

[CR23] Gunst S, Zhang W (2008). Actin cytoskeletal dynamics in smooth muscle: a new paradigm for the regulation of smooth muscle contraction. Am J Physiol Cell Physiol.

[CR24] Hai CM, Murphy RA (1988). Cross-bridge phosphorylation and regulation of latch state in smooth muscle. Am J Physiol.

[CR25] Hansen N (2016) The CMA evolution strategy: a tutorial. arXiv 1604.00772

[CR26] Hansen N (2009). A method for handling uncertainty in evolutionary optimization with an application to feedback control of combustion. IEEE Trans Evol Comput.

[CR27] Harder D (1984). Pressure-dependent membrane depolarization in cat middle cerebral artery. Circ Res.

[CR28] Harraz OF et al (2022) Piezo1 is a mechanosensor channel in central nervous system capillaries. Circ Res 130(10):1531–1546 10.1161/CIRCRESAHA.122.320827 PMC9106929 35382561

[CR29] Irons L, Humphrey J (2020). Cell signaling model for arterial mechanobiology. PLoS Comput Biol.

[CR30] Irons L, Latorre M, Humphrey J (2021). From transcript to tissue: multiscale modeling from cell signaling to matrix remodeling. Ann Biomed Eng.

[CR31] Jackson W (2021). Calcium-dependent ion channels and the regulation of arteriolar myogenic tone. Front Physiol.

[CR32] Johnson R et al (2009) Ca^2+^ sensitization via phosphorylation of myosin phosphatase targeting subunit at threonine-855 by Rho kinase contributes to the arterial myogenic response. J Physiol 587(11):2537–2553 10.1113/jphysiol.2008.168252 PMC2714019 19359365

[CR33] Kapela A et al (2008) A mathematical model of Ca^2+^ dynamics in rat mesenteric smooth muscle cell: agonist and no stimulation. J Theor Biol 253:238–260 10.1016/j.jtbi.2008.03.004 18423672

[CR34] Knot H, Nelson M (1998). Regulation of arterial diameter and wall [Ca^2+^] in cerebral arteries of rat by membrane potential and intravascular. J Physiol.

[CR35] Lagaud G (2002). Pressure-dependent myogenic constriction of cerebral arteries occurs independently of voltage-dependent activation. Am J Physiol Heart Circ Physiol.

[CR36] Li Y, Brayden J (2017). Rho kinase activity governs arteriolar myogenic depolarization. J Cereb Blood Flow Metab.

[CR37] Lidington A, Schubert R, Bolz S-S (2013). Capitalizing on diversity: an integrative approach towards the multiplicity of cellular mechanisms underlying myogenic responsiveness. Cardiovasc Res.

[CR38] Mederos Y, Schnitzler M (2008). Gq-coupled receptors as mechanosensors mediating myogenic vasoconstriction. EMBO J.

[CR39] Monson K (2008). Biaxial response of passive human cerebral arteries. Ann Biomed Eng.

[CR40] Moreno-Dominguez A et al (2014) Cytoskeletal reorganization evoked by Rho-associated kinase- and protein kinase C-catalyzed phosphorylation of cofilin and heat shock protein 27, respectively, contributes to myogenic constriction of rat cerebral arteries. J Biol Chem 289(30):20939–20952 10.1074/jbc.M114.553743 PMC4110300 24914207

[CR41] Mufti R (2010). Intravascular pressure augments cerebral arterial constriction by inducing voltage-insensitive Ca^2+^ waves. J Physiol.

[CR42] Murtada S-I (2010). A calcium-driven mechanochemical model for prediction of force generation in smooth muscle. Biomech Model Mechanobiol.

[CR43] Murtada S-I (2012). Experiments and mechanochemical modeling of smooth muscle contraction: significance of filament overlap. J Theor Biol.

[CR44] Murtada S-I, Holzapfel GA (2014). Investigating the role of smooth muscle cells in large elastic arteries: a finite element analysis. J Theor Biol.

[CR45] Murtada S-I, Lewin S, Arner A, Humphrey J (2016). Adaptation of active tone in the mouse descending thoracic aorta under acute changes in loading. Biomech Model Mechanobiol.

[CR46] Osol G et al (2002) Myogenic tone, reactivity, and forced dilatation: a three-phase model of in vitro arterial myogenic behavior. Am J Physiol Heart Circ Physiol 283:H2260–H2267 10.1152/ajpheart.00634.2002 12388265

[CR47] Pires P et al (2017) The angiotensin II receptor type 1b is the primary sensor of intraluminal pressure in cerebral artery smooth muscle cells. J Physiol 595(14):4735–4753 10.1113/JP274310 PMC5509855 28475214

[CR48] Schubert R, Lidington D, Bolz S-S (2008) The emerging role of Ca^2+^ sensitivity regulation in promoting myogenic vasoconstriction. Cardiovasc Res 77(1):8–18 10.1016/j.cardiores.2007.07.018 17764667

[CR49] Walsh M, William W (2013). The role of actin filament dynamics in the myogenic response of cerebral resistance arteries. J Cereb Blood Flow Metab.

[CR50] Yang J (2003). The myogenic response in isolated rat cerebrovascular arteries: smooth muscle cell model. Med Eng Phys.

[CR51] Yang J (2003). The myogenic response in isolated rat cerebrovascular arteries: vessel model. Med Eng Phys.

