# Correction to: A new model for evaluating pressure‑induced vascular tone in small cerebral arteries

**DOI:** 10.1007/s10237-023-01795-2

**Published:** 2024-02-15

**Authors:** Alberto Coccarelli, Sanjay Pant, Ioannis Polydoros, Osama F. Harraz

**Affiliations:** 1 Zienkiewicz Institute for Modelling, Data and AI, Faculty of Science and Engineering, Swansea University, Swansea, UK; 2 Department of Pharmacology, Larner College of Medicine, and Vermont Center for Cardiovascular and Brain Health, University of Vermont, Burlington, USA

**Correction to: Biomechanics and Modeling in Mechanobiology** 10.1007/s10237-023-01774-7

The superscript 'opt' in baruopt_fs was incorrectly fixed in the second-to-last sentence of section 3.2 of the original online version of this article; the amended text is baru_fs is corrected with this correction.

The original article has been corrected.

